# HEXACO Personality Traits and Risky Choices in Hypothetical Financial Lotteries: A Repeated-Measures Study

**DOI:** 10.3390/bs16081389

**Published:** 2026-08-12

**Authors:** Bartosz Wiszniewski, Hanna Liberska

**Affiliations:** 1 Faculty of Psychology, Kazimierz Wielki University, 85-064 Bydgoszcz, Poland; hanna.liberska@op.pl; 2 Clinical Hospital of Emili Warmiński, 85-064 Bydgoszcz, Poland; 3 Institute of Psychology, University of Zielona Góra, 65-417 Zielona Gora, Poland

**Keywords:** risk choice, HEXACO, prospect theory, Honesty–Humility, conscientiousness, hypothetical financial lotteries

## Abstract

Risky choice depends not only on individual differences but also on characteristics of the decision context, including how decision problems are framed. This study examined whether HEXACO personality dimensions were associated with risky choices in four hypothetical financial lotteries varying by outcome domain and stake size. A total of 310 Polish adults completed the HEXACO-60 inventory and made decisions in lotteries involving small gains, large gains, small losses, and large losses. Lottery type was strongly associated with choice: participants more often selected the safe option in gain conditions and the risky option in loss conditions. In contrast, only Honesty–Humility showed a significant overall association with risky choice after controlling for lottery type, whereas the remaining HEXACO dimensions did not. Interaction analyses further indicated that the associations of Honesty–Humility and Conscientiousness with risky choice varied across lottery scenarios. Higher Honesty–Humility was associated with lower odds of choosing the risky option in gain lotteries, whereas higher Conscientiousness was associated with higher odds of choosing the risky option in loss lotteries. These findings provide preliminary evidence that selected personality traits show context-dependent associations with risky choices across the four hypothetical lottery scenarios examined in this study. However, because the study used only four hypothetical scenarios, including two involving very large monetary amounts, these effects should be interpreted cautiously and require replication using broader and incentive-compatible measures of financial risk taking.

## 1. Introduction

Risk taking has long been studied as a relatively stable individual disposition in both personality psychology and decision-making research. However, accumulating evidence indicates that risk taking is not a fully domain-general tendency, but also depends on the characteristics of the decision context, including the domain of choice and the way potential outcomes are framed. Contemporary approaches therefore view risky choice as the result of interactions between situational and individual factors, among which personality traits may play an important role (Blais & Weber, 2006; Hanoch et al., 2006; Tyszka & Domurat, 2004; Weber et al., 2002). This perspective has also been supported by recent research. For example, Balasuriya et al. (2025) showed that personality traits interacted with prior financial outcomes in predicting subsequent risk-taking in a sequential investment simulation, suggesting that personality–risk associations may depend on situational characteristics and previous decision outcomes rather than reflecting stable, context-independent tendencies.

One of the most influential frameworks for understanding decisions under risk is prospect theory. Kahneman and Tversky showed that human choices systematically deviate from classical normative models and depend strongly on whether outcomes are framed as gains or losses. Later research extended this perspective by emphasizing that individual differences, including personality traits, may shape preferences for safe versus risky options (Kahneman & Tversky, 1979; Nicholson et al., 2005). Previous studies on personality and risk taking have focused primarily on the Big Five model. In comparison, less is known about how traits from the HEXACO model are associated with risky choices across different types of financial decision scenarios.

The present study examined whether HEXACO personality traits were associated with the probability of selecting the risky option in four hypothetical financial lotteries constructed according to the logic of prospect theory. The lotteries represented small gains, large gains, small losses, and large losses. Rather than attempting to establish a comprehensive model of financial risk taking, the present study was designed to examine whether previously reported associations between HEXACO personality traits and risky choice can be replicated across four specific hypothetical lottery scenarios differing in outcome domain and stake size. This design allowed comparisons of participants’ responses across the four specific decision scenarios and enabled examination of whether personality–choice associations differed between them.

### 1.1. Domain Specificity of Risk

Contemporary research increasingly rejects the view of risk taking as a homogeneous, domain-general individual characteristic. Evidence shows that individuals may respond to risk differently across domains and situations (MacCrimmon & Wehrung, 1986; Schoemaker, 1990). Accordingly, domain-specific approaches assume that risk propensity depends on the area of functioning and on the characteristics of the particular decision context. For example, the same person may be willing to take financial risks while avoiding risk in health-related or social domains. Risky decisions should therefore be analysed not only as manifestations of relatively stable dispositions, but also as a function of how the decision problem is represented, what options are available, and the context in which the choice is made (Blais & Weber, 2006; Bromiley & Curley, 1992; MacCrimmon & Wehrung, 1986; Schoemaker, 1990).

Weber et al. developed the Domain-Specific Risk-Taking Scale (DOSPERT), which assesses risk taking across commonly studied domains, including social, recreational, investment, gambling, health/safety, and ethical risk (Weber et al., 2002). Hanoch et al. found that extreme sports enthusiasts were more likely than others to take recreational risks, such as skydiving or kayaking, but were not especially prone to health-related or financial risks. Similarly, smokers were more likely to take health-related risks, but did not differ from non-smokers in their propensity to take risks in other domains (Hanoch et al., 2006). Together, these findings indicate that risk taking is a multifaceted construct shaped by both dispositional and contextual factors.

This perspective is consistent with behavioural economics, which assumes that risky decisions depend not only on objective choice parameters, but also on the psychological representation of the decision problem (Godłów-Legiędź, 2013; Solek, 2010).

### 1.2. Financial Decisions in Prospect Theory

Kahneman and Tversky’s prospect theory is one of the most influential frameworks for explaining decisions under risk. It assumes that people evaluate decision outcomes not in terms of their final level of wealth, but relative to a reference point, and that possible outcomes are interpreted as gains or losses. The value function is concave for gains and convex for losses, and is steeper in the domain of losses, reflecting loss aversion. In financial decision making, these assumptions imply that risky choice should depend on how outcomes are framed. In the domain of gains, people are more likely to choose the certain option. This pattern is referred to as the certainty effect and reflects the tendency to overweight certain outcomes relative to merely probable ones. In the domain of losses, people are more likely to accept risk, especially when the risky option offers a chance to avoid a certain loss. This pattern is referred to as the reflection effect (Kahneman & Tversky, 1979).

Prospect theory also emphasizes the role of decision weights, that is, the subjective transformation of probabilities. Financial decisions therefore depend not only on the value of potential gains and losses, but also on how probabilities are psychologically weighted (Kahneman & Tversky, 1979; Zielonka, 2005).

In the present study, four hypothetical financial lotteries were used: small gains, large gains, small losses, and large losses. The design made it possible to examine whether the frequency of selecting the risky option differed across these four scenarios. It also allowed us to test whether the associations between HEXACO personality traits and risky choice differed between the specific lottery conditions.

### 1.3. Personality and Risky Choice Across Decision Contexts

Numerous studies have shown that risk taking is associated with personality traits, although the strength and direction of these associations vary depending on the behavioural domain and the method used to measure risk (Breakwell, 2014; Nicholson et al., 2005; Sekścińska & Markiewicz, 2020). Most previous research has focused on the Big Five model, whereas the HEXACO model has received less attention in this context despite its potential explanatory value. A distinctive feature of the HEXACO model is the Honesty–Humility dimension, which includes sincerity, fairness, modesty, and a low tendency to exploit others for personal gain. Ashton and Lee argued that these characteristics are best conceptualized as a separate factor because they account for unique variance related to the avoidance of dishonest behaviour (Ashton & Lee, 2007). Honesty–Humility has no direct equivalent in the classic Big Five model and may be particularly relevant to financial decisions and profit-oriented behaviour.

Previous research indicates that lower Honesty–Humility is associated with a greater willingness to engage in risky behaviours, including financial risk taking (de Vries et al., 2009; Weller & Thulin, 2012; Weller & Tikir, 2011). Individuals low in this trait may focus more strongly on potential benefits while being less attentive to possible losses and threats (Weller & Tikir, 2011). These findings provide a basis for examining whether Honesty–Humility is associated with the probability of selecting risky options in financial lottery tasks. Similar conclusions have been drawn from studies on gambling behaviour, in which lower Honesty–Humility was associated with greater involvement in activities aimed at quick gains despite uncertain consequences (Kim et al., 2018).

Other personality dimensions may also be linked to risky choice through distinct mechanisms of behavioural regulation. Higher Emotionality is often associated with greater threat perception, stronger responses to potential loss, and a more cautious decision-making style, which may reduce the likelihood of choosing risky options (Weller & Thulin, 2012; Weller & Tikir, 2011). Extraversion is more often linked to activity, stimulation seeking, and reward orientation, and may therefore be positively associated with risk acceptance, especially in financial and investment contexts (Sekścińska & Markiewicz, 2020). Openness to Experience may foster tolerance of uncertainty and willingness to consider novel or less conventional options, which has been linked in some studies to greater risk acceptance and interest in non-standard financial activities (Sekścińska & Markiewicz, 2020; Sekścińska et al., 2022). Agreeableness may function as a protective factor, reflecting greater caution, sensitivity to the consequences of decisions, and a lower tendency to engage in uncertain actions (Nicholson et al., 2005; Sekścińska et al., 2022). The role of Conscientiousness appears more complex. On the one hand, it is associated with planning, self-control, and deliberation, which may limit risky choices. On the other hand, some studies suggest that its relationship with risk may depend on the nature of the task and on whether the decision involves gains or losses (Nicholson et al., 2005; Weller & Thulin, 2012; Weller & Tikir, 2011).

Taken together, these findings suggest that personality may influence risky decisions through several partially distinct mechanisms, including threat perception, sensitivity to potential losses, reward orientation, self-control, and tolerance of uncertainty. At the same time, current evidence does not support treating these associations as simple or universal. Their strength may vary depending on whether a decision involves potential gains or losses, as well as on the characteristics of the decision-making task. This conclusion is also consistent with recent literature reviews, which indicate that while Extraversion shows relatively consistent positive associations with risk taking, the effects of the remaining personality traits vary substantially depending on the behavioural domain, measurement approach, and decision context (Moncel et al., 2025). However, although previous studies have demonstrated associations between personality traits and financial risk taking, it remains unclear whether these associations remain stable across systematically varied financial decision contexts within a common experimental paradigm. The present study therefore examines whether HEXACO personality traits are associated with the probability of selecting the risky option and whether these associations remain consistent or differ across four structurally comparable hypothetical financial lotteries that systematically vary gain–loss framing and stake magnitude. By examining these contextual manipulations within a single repeated-measures experimental paradigm, the study provides an opportunity to evaluate whether personality–risk associations are relatively stable or depend on the characteristics of the decision context.

### 1.4. Rationale for the Research Model

The theoretical assumptions outlined above suggest that responses to financial lottery choices may depend on both the characteristics of the decision scenario and person-level characteristics. From the perspective of prospect theory, key contextual factors include whether outcomes are framed as gains or losses and the magnitude of the stake. From the perspective of individual-differences research, personality traits may influence risky choice through mechanisms such as threat perception, reward orientation, self-control, and tolerance of uncertainty. Accordingly, the present study examined both the direct effects of lottery type and HEXACO personality traits on risky choice and the interactions between personality traits and lottery type (Kahneman & Tversky, 1979; Nicholson et al., 2005).

The following hypotheses were formulated:

**Hypothesis** **1 (H1).** *The probability of selecting the risky option differs across the four hypothetical lottery scenarios: ALFA, BETA, GAMMA, and DELTA*.

**Hypothesis 2** **(H2).** *HEXACO personality dimensions are associated with the probability of selecting the risky option across the four hypothetical lottery scenarios, after controlling for lottery condition*.

**Hypothesis 3** **(H3).** *The associations between HEXACO dimensions and the probability of selecting the risky option vary across the four hypothetical lottery scenarios, as reflected in HEXACO × Lottery interactions*.

**Figure 1:**
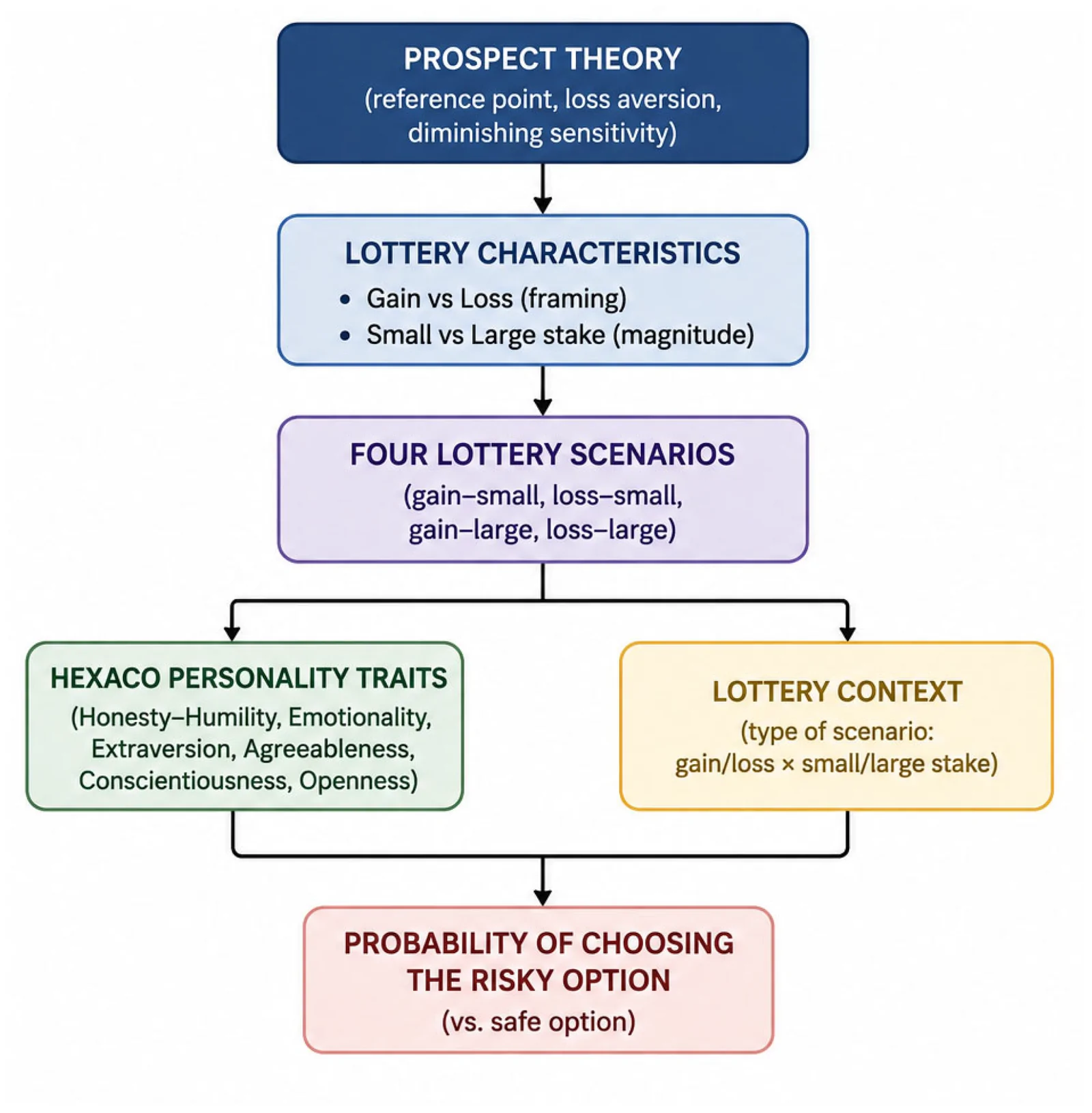
Theoretical framework of the study.

The theoretical framework underlying the present study is presented in Figure 1.

Based on previous research, it was expected that personality traits related to lower caution, stronger reward orientation, lower threat sensitivity, or greater tolerance of uncertainty would be associated with a greater likelihood of choosing the risky option. However, because previous findings do not provide strong grounds for precise trait-specific predictions across each of the four lottery conditions, the interaction analyses were treated as context-sensitive tests of whether personality–risk associations varied between decision scenarios.

## 2. Materials and Methods

### 2.1. Participants

The study included 310 adult Polish citizens. The sample consisted of 163 women and 147 men. Participants’ mean age was 45.49 years (SD = 16.50), with an age range from 18 to 92 years.

Participants were recruited through the nationwide Ariadna research panel. This recruitment procedure allowed for a sample diverse in basic sociodemographic characteristics, including gender, age, place of residence, education level, and financial situation. The inclusion criteria were adulthood, Polish citizenship, and fluency in Polish.

Each participant completed four lottery tasks, resulting in a total of 1240 decision-level observations. Because the study used a repeated-measures design, decisions were nested within participants. This nested data structure was accounted for in the statistical analyses using mixed-effects logistic regression models.

### 2.2. Procedure

The study was conducted online in Polish. Before participation, all individuals provided informed consent. Participants were asked to choose the option that best reflected their preference in each decision-making task. The four lottery tasks were presented in random order. The average completion time was approximately 20 min.

### 2.3. Measures

HEXACO-60

Personality traits were measured using the Polish version of the HEXACO-60 inventory (Ashton & Lee, 2009; Skimina et al., 2020), a shortened version of the HEXACO Personality Inventory-Revised. The instrument measures six basic personality dimensions: Honesty-Humility, Emotionality, Extraversion, Agreeableness, Conscientiousness, and Openness to Experience. The questionnaire consists of 60 items, with 10 items for each domain scale. Responses are given on a 5-point Likert scale ranging from 1 (“strongly disagree”) to 5 (“strongly agree”). In the present sample, scale reliability was assessed using Cronbach’s alpha. The obtained values were as follows: Honesty–Humility α = 0.74, Emotionality α = 0.70, Extraversion α = 0.73, Agreeableness α = 0.67, Conscientiousness α = 0.75, and Openness to Experience α = 0.70.

Although the internal consistency of Agreeableness (α = 0.67) was slightly below the conventional threshold of 0.70, it was deemed acceptable for the purposes of the present study. Nevertheless, the relatively lower reliability of this scale may have attenuated associations involving Agreeableness, potentially reducing the likelihood of detecting significant effects. Therefore, findings related to this dimension should be interpreted with appropriate caution.

Financial lottery tasks

The dependent variable was measured using four hypothetical financial lottery tasks. Each task required participants to choose between a safe option and a risky option. The safe option represented a certain gain or a certain loss, whereas the risky option involved a specified probability of obtaining a larger gain, incurring a larger loss, or avoiding a financial loss.

The tasks were constructed in line with the assumptions of prospect theory. Two lotteries involved potential gains and two involved potential losses. The lotteries also differed in stake size. The ALFA lottery involved small gains, the GAMMA lottery involved large gains, the BETA lottery involved small losses, and the DELTA lottery involved large losses. In each lottery, choosing the risky option was coded as 1, and choosing the safe option was coded as 0.

Because each decision context was represented by a single lottery task, the measure should be interpreted as an assessment of risky choice in four specific hypothetical scenarios rather than as a comprehensive measure of financial risk taking.

The four lotteries used in the study were as follows:

ALFA lottery:
0: A certain gain of PLN 3000
1: An 80% chance of gaining PLN 4000 and a 20% chance of gaining nothing
BETA lottery:
0: A certain loss of PLN 3000
1: An 80% chance of losing PLN 4000 and a 20% chance of no loss
GAMMA lottery:
0: A certain gain of PLN 300,000
1: An 80% chance of gaining PLN 400,000 and a 20% chance of gaining nothing
DELTA lottery:
0: A certain loss of PLN 300,000
1: An 80% chance of losing PLN 400,000 and a 20% chance of no loss

The safe and risky options were intentionally constructed with slightly different expected values. In the gain lotteries, the expected value of the risky option exceeded that of the certain option (PLN 3200 vs. PLN 3000; PLN 320,000 vs. PLN 300,000). In the loss lotteries, the risky option was associated with a larger expected loss (−PLN 3200 vs. −PLN 3000; −PLN 320,000 vs. −PLN 300,000). Consequently, participants’ choices may have reflected responses to both outcome uncertainty and expected-value differences. Therefore, the dependent variable should be interpreted as the choice between the specific alternatives presented rather than as a process-pure measure of risk preference.

The inclusion of two stake sizes was intended to increase the contrast in the magnitude of potential outcomes while preserving the same probability structure and proportional relationship between the certain and risky options. The large-stake lotteries were therefore not designed to simulate typical everyday financial decisions but to examine whether personality–choice associations remained similar when the nominal consequences of the decision were substantially increased.

### 2.4. Data Analysis Strategy

The main inferential analyses were conducted using generalized linear mixed models (GLMMs) with a binomial family and a logit link function. The dependent variable was the choice made in each decision-making task, coded as 1 for the risky option and 0 for the safe option. The use of mixed-effects models was justified by the repeated-measures structure of the data, as each participant made decisions in four lottery scenarios. The models accounted for the nesting of observations within participants by including a random intercept for participant ID (1|ID). A more complex random-effects structure including random slopes was not adopted because each participant contributed only four repeated observations. Under these conditions, a random-intercept model was considered sufficient to account for the repeated-measures design while providing stable parameter estimation. Analyses were conducted on data in long format, comprising 1240 observations from 310 participants.

The decision context was represented by the Lottery factor, which included four conditions: ALFA (small gains), GAMMA (large gains), BETA (small losses), and DELTA (large losses). Lottery was analyzed as a four-level within-subject factor rather than as separate gain/loss and stake-size factors because each combination of outcome domain and stake size was represented by only one specific lottery scenario. Reference coding was used for this factor, with ALFA serving as the reference category. Therefore, the remaining parameters for the Lottery factor were interpreted as differences from the ALFA condition.

Continuous predictors, including HEXACO dimensions, were standardized. This allowed the regression coefficients to be interpreted as changes in the log-odds of choosing the risky option associated with a one standard deviation increase in the predictor.

A single generalized linear mixed-effects model was estimated that included all six HEXACO personality dimensions, lottery type, and all HEXACO × Lottery interaction terms simultaneously. This approach allowed the unique contribution of each personality dimension to be evaluated while controlling for the remaining HEXACO traits.

The significance of fixed effects (main effects and interactions) was assessed using Type III Wald χ^2^ tests. Regression coefficients (b), standard errors (SE), Wald t-statistics, *p*-values, and odds ratios (OR = exp(b)) were reported for the fixed effects included in the model.

Following significant omnibus interaction tests, simple-effect analyses were conducted by successively changing the reference category of the Lottery factor. This procedure allowed the association between each HEXACO dimension and risky choice to be estimated separately within each lottery condition.

Regression coefficients were interpreted on the logit scale, that is, as log-odds. To facilitate interpretation, they were converted into odds ratios using the formula OR = exp(b). OR values indicate how much the odds of choosing the risky option change with a one standard deviation increase in the predictor, holding other variables constant. For the Lottery factor, OR values refer to comparisons between a given lottery condition and the reference condition.

Because multiple tests were conducted, the Benjamini–Hochberg false discovery rate correction was applied (FDR; q = 0.05). The correction was performed separately for families of tests involving the main effects of HEXACO dimensions, interactions between HEXACO dimensions and lottery type, and simple effects in individual lottery conditions.

All statistical tests were two-tailed, and the significance level was set at α = 0.05. Because the dependent variable was binary, the assumption of normality of the dependent variable was not applicable; therefore, models with a binomial family and a logit link function were used. All statistical analyses were performed using JASP software version 0.19.3.

Use of generative artificial intelligence tools

During the preparation of this manuscript, the authors used ChatGPT, (OpenAI, GPT-5.5) a large language model developed by OpenAI, to support language editing, text organization, and stylistic refinement. The tool was not used to generate original data, conduct statistical analyses, or make scientific conclusions. All content, analyses, interpretations, and final decisions regarding the manuscript were reviewed and approved by the authors, who take full responsibility for the submitted work.

## 3. Results

First, the distribution of safe and risky choices was examined for each lottery. Table 1 shows the frequencies and percentages of choices in the four lottery conditions: ALFA, BETA, GAMMA, and DELTA.

The distribution of responses indicated clear differences between gain and loss lotteries. In the gain lotteries, safe choices were predominant: 74.5% of participants chose the safe option in ALFA and 79.7% in GAMMA. A different pattern was observed in the loss lotteries, where the risky option was chosen more frequently: 54.5% of participants chose the risky option in BETA and 58.4% in DELTA. These results are consistent with prospect theory, according to which individuals are more likely to avoid risk in the gain domain and more likely to take risk in the loss domain.

**Verification of Hypothesis 1**

To test H1, a generalized linear mixed model was estimated with risky choice as the dependent variable and lottery type as the fixed predictor. A random intercept for participant was included to account for the non-independence of repeated observations within participants. Model fit statistics are presented in Table 2.

The GLMM with a random intercept for participant showed a significant effect of lottery type on risky choice, χ^2^(3) = 206.795, *p* < 0.001 (Table 3). Compared with the reference condition, ALFA, the odds of choosing the risky option were significantly higher in BETA, b = 1.862, SE = 0.226, *p* < 0.001, OR = 6.44, and in DELTA, b = 2.100, SE = 0.231, *p* < 0.001, OR = 8.17. Thus, the odds of risky choice were approximately 6.4 times higher in BETA and 8.2 times higher in DELTA than in ALFA. In contrast, the comparison between the two gain lotteries (ALFA vs. GAMMA) was not statistically significant, b = −0.411, SE = 0.228, *p* = 0.072, OR = 0.66, indicating that participants’ likelihood of choosing the risky option did not differ reliably between these two gain scenarios. Overall, these findings support H1 by showing that the probability of selecting the risky option differed significantly across the four hypothetical lottery scenarios, although the absence of a significant difference between ALFA and GAMMA suggests that differences in stake size within the gain domain may have exerted a weaker influence on choice than differences between gain and loss contexts.

**Verification of Hypothesis 2**

After establishing that risky choice differed across lottery types, we tested whether HEXACO personality traits were associated with risky choice after controlling for lottery type. For this purpose, a generalized linear mixed model was estimated in which lottery type and the six HEXACO personality dimensions were included as fixed predictors. Model fit statistics are presented in Table 4.

A significant main effect was observed for Honesty–Humility, χ^2^(1) = 8.131, *p* = 0.004, *p* FDR = 0.024. Higher levels of Honesty–Humility were associated with a lower overall probability of selecting the risky option (b = –0.632, SE = 0.222, *p* = 0.004, OR = 0.53). No significant main effects were found for Emotionality, Extraversion, Agreeableness, Conscientiousness, or Openness to Experience (Table 5). However, because the Honesty–Humility × Lottery interaction was also significant (Table 6), this overall association should be interpreted with caution, as it varied across lottery conditions.

Thus, H2 was only partially supported. Although no general effects were observed for most HEXACO dimensions, Honesty–Humility showed a significant overall association with the probability of selecting the risky option, qualified by a significant interaction with lottery type.

**Verification of Hypothesis 3**

In the next step, GLMMs including interactions between individual HEXACO dimensions and lottery type were estimated. These analyses tested whether the associations between personality traits and risky choice differed across decision conditions (Table 6).

Table 6 presents the results of the omnibus tests of interactions between HEXACO dimensions and lottery type in the generalized linear mixed-effects model (GLMM). After applying the Benjamini–Hochberg correction, the Honesty-Humility × Lottery and Conscientiousness × Lottery interactions remained statistically significant. This indicates that the associations of Honesty-Humility and Conscientiousness with risky choice varied depending on lottery type. The nature of the significant interactions was further examined using simple effects analyses, presented in Table 7. Overall, these results partially support H3 by showing that the associations between selected HEXACO traits and the probability of selecting the risky option differed across the four lottery scenarios.

Because the interactions of Honesty-Humility and Conscientiousness with lottery type were significant, simple effects analyses were conducted. These analyses examined the lottery conditions under which each personality trait was associated with risky choice. The simple effects of Honesty-Humility and Conscientiousness within each lottery condition are presented in Table 7.

The simple effects analyses presented in Table 7 showed that Honesty–Humility was significantly associated with lower odds of choosing the risky option in the gain lotteries, ALFA and GAMMA. In BETA, the effect was positive but did not remain statistically significant after FDR correction. In DELTA, the effect was not statistically significant. For Conscientiousness, significant positive effects were found in the loss lotteries, BETA and DELTA, indicating that higher Conscientiousness was associated with higher odds of choosing the risky option under loss conditions. The effect of Conscientiousness in ALFA was negative but did not remain statistically significant after FDR correction, whereas no significant effect was found in GAMMA. Overall, these findings provide partial support for H3, as the associations of Honesty–Humility and Conscientiousness with risky choice varied across lottery conditions. The association observed for Conscientiousness in loss lotteries was opposite to the expected general direction and should therefore be interpreted as an unexpected context-dependent finding.

**Figure 2:**
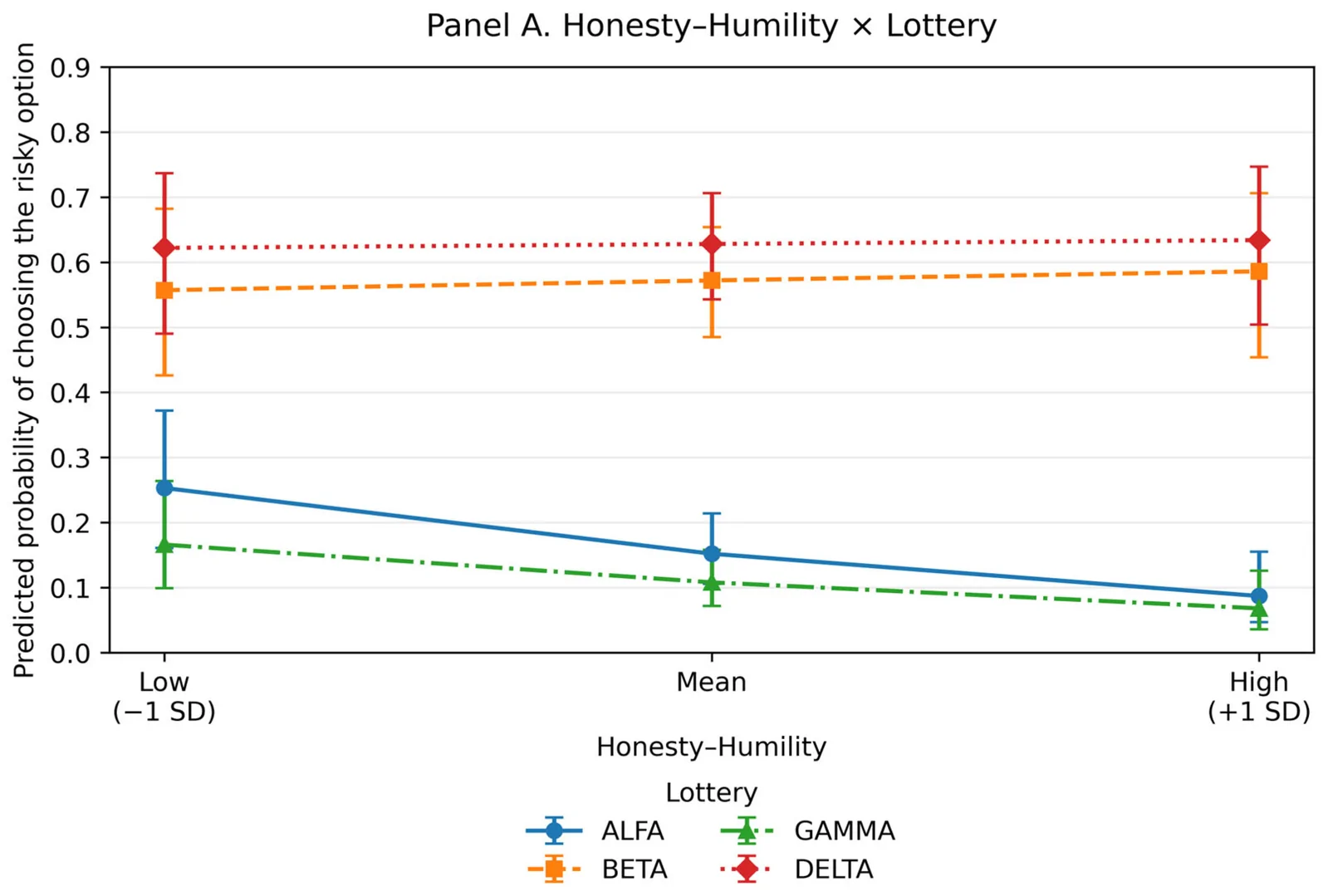

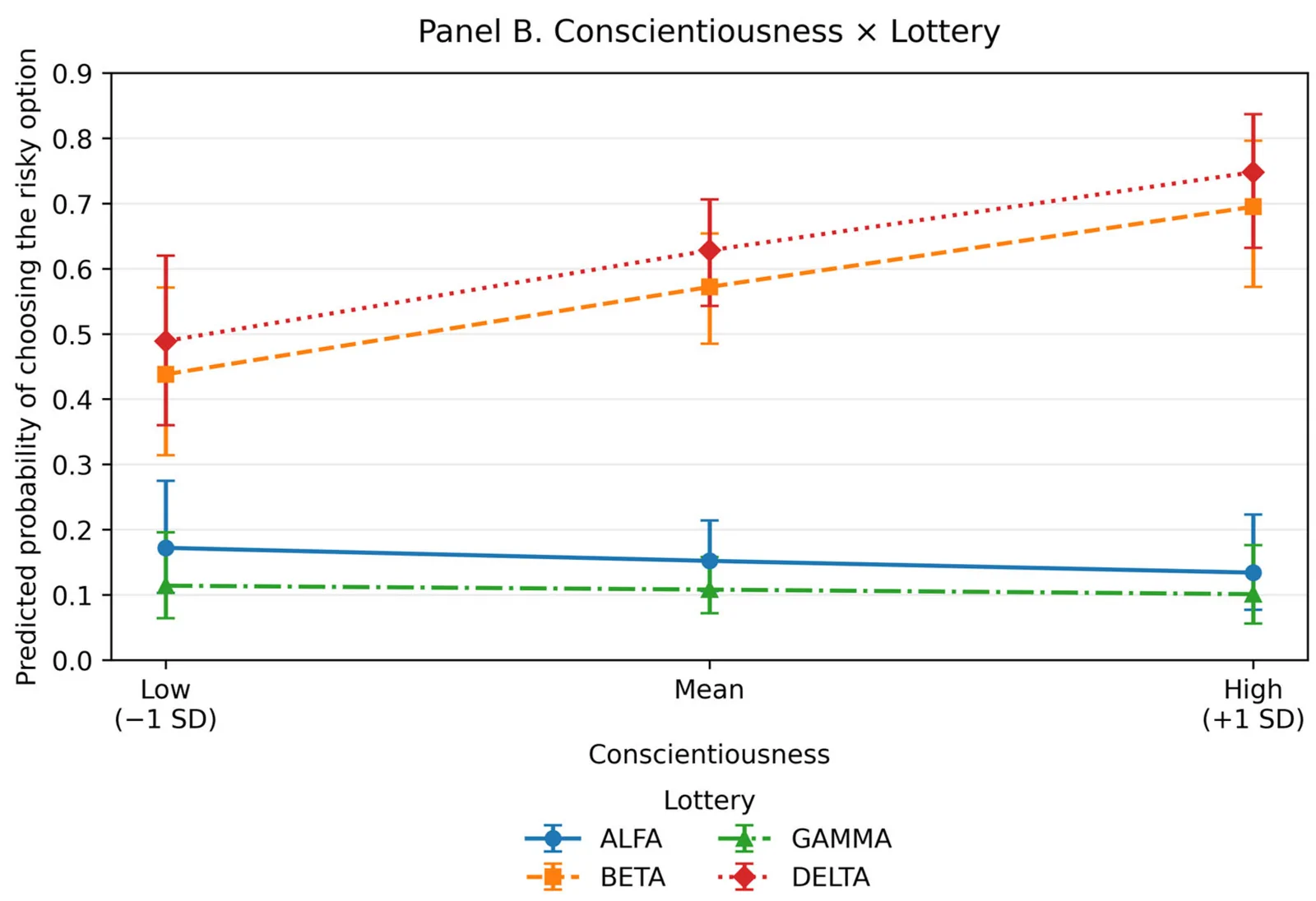
Estimated predicted probabilities of selecting the risky option across lottery conditions at low (−1 SD), mean, and high (+1 SD) levels of Honesty–Humility (**A**) and Conscientiousness (**B**). Error bars indicate 95% confidence intervals.

To facilitate interpretation of the significant interaction effects, Table 8 presents estimated predicted probabilities on the response scale together with their 95% confidence intervals, whereas Figure 2 provides a graphical representation of these interaction effects across lottery conditions.

## 4. Discussion

The present study examined whether the probability of selecting the risky option differed across four hypothetical financial lottery scenarios and whether HEXACO personality traits were associated with these choices. Particular attention was given to whether the associations between HEXACO dimensions and risky choice depended on decision context, specifically gain versus loss framing and stake size. The results showed that lottery type strongly differentiated participants’ decisions. Among the HEXACO dimensions, only Honesty–Humility showed a significant overall association with risky choice, whereas the remaining personality traits did not emerge as general predictors. In addition, selected personality traits showed context-dependent associations with risky choice in specific lottery conditions. These effects should be interpreted cautiously, given the limited number of hypothetical decision scenarios used in the study.

Consistent with H1, risky choice differed across lottery conditions. Participants were more likely to choose the safe option in gain lotteries and the risky option in loss lotteries. Compared with the ALFA condition, the odds of choosing the risky option were significantly higher in BETA and DELTA, both of which involved potential losses. This pattern is consistent with prospect theory, according to which individuals tend to avoid risk in the domain of gains but become more willing to accept risk in the domain of losses, particularly when the risky option offers a chance to avoid a certain loss (Kahneman & Tversky, 1979). At the same time, because each condition was represented by a single lottery scenario, these findings should be interpreted as evidence of differences between the specific lottery conditions used in the study rather than as a comprehensive test of gain, loss, and stake-size effects. More generally, the observed response patterns are consistent with the view that risky choices may vary across decision contexts rather than reflecting only a stable, context-independent disposition. However, the present design does not allow conclusions about participants’ broader or stable risk preferences. (Blais & Weber, 2006; Hanoch et al., 2006; Weber et al., 2002).

An additional issue concerns the interpretation of the dependent variable. Because the risky and safe options differed slightly in expected value, participants’ choices cannot be attributed exclusively to risk preference. In the gain lotteries, the risky option offered a higher expected payoff, whereas in the loss lotteries it involved a larger expected loss. Consequently, the observed choices may have reflected a combination of responses to outcome uncertainty, expected-value differences, and framing effects. The present findings should therefore be interpreted as referring to participants’ responses to the specific lottery scenarios rather than as evidence of general risk preferences.

The absence of a significant difference between the two gain lotteries suggests that, within the present set of lottery scenarios, differences in stake size may have had a weaker influence on participants’ choices than differences between gain and loss contexts. However, because the study was not designed as a factorial manipulation of framing and stake size and each condition was represented by a single lottery, this interpretation should be regarded as tentative.

H2 was partially supported. Honesty–Humility showed a significant overall association with the probability of selecting the risky option after controlling for lottery type, whereas the remaining HEXACO dimensions did not. However, because the association between Honesty–Humility and risky choice also varied across lottery conditions, as indicated by the significant Honesty–Humility × Lottery interaction, this overall effect should be interpreted in conjunction with the interaction analyses rather than as a uniform association across all decision contexts. This finding indicates that, within the four lottery scenarios used in the present study, personality did not show a general association with the probability of selecting the risky option. Rather, the associations between personality and risky choice may depend on the behavioural domain, the method used to assess risk, and the characteristics of the decision-making situation (Blais & Weber, 2006; Hanoch et al., 2006; Weber et al., 2002). The absence of significant general effects indicates that the present findings do not support interpreting HEXACO traits as broad predictors of financial risk taking beyond the four hypothetical lottery scenarios examined here.

H3 was partially supported. Significant interactions with lottery type were found for Honesty–Humility and Conscientiousness, indicating that the associations of these traits with risky choice differed across decision contexts. Importantly, these interaction effects remained significant when all six HEXACO dimensions and their interactions with lottery type were estimated simultaneously within a single generalized linear mixed-effects model, supporting the robustness of these context-dependent associations. Higher Honesty–Humility was associated with lower odds of choosing the risky option in the ALFA and GAMMA gain lotteries. This finding is partially consistent with previous studies showing that lower Honesty–Humility is associated with greater willingness to engage in risky behaviours, including financial risk taking (de Vries et al., 2009; Weller & Thulin, 2012; Weller & Tikir, 2011). However, whereas Weller and Thulin (2012) reported that lower Honesty–Humility was associated with greater risk-taking across both gain and loss contexts, the present findings indicate a more context-specific pattern, with the association emerging only in gain lotteries. This discrepancy may reflect methodological differences, including the number and structure of lottery scenarios, as well as differences in the operationalization of risky decision-making. One possible interpretation is that individuals higher in Honesty–Humility may be less strongly oriented toward maximizing potential gains and therefore less likely to choose a risky option when a certain gain is available. However, this interpretation remains tentative, because the present study did not directly measure gain-maximization motives, perceived attractiveness of the risky option, or subjective evaluations of the lotteries. Therefore, this result should be treated as a context-specific association that requires replication in studies using a broader set of gain-related financial decision tasks.

Although gambling differs from hypothetical lottery decisions in several important respects, previous research has also linked lower Honesty–Humility to greater gambling involvement and gambling severity (Kim et al., 2018). Considered together with the present findings, this pattern suggests that Honesty–Humility may represent a relatively consistent correlate of financial risk-related behaviour across different decision-making contexts. At the same time, the differences observed between gambling studies and the present lottery task indicate that the strength and expression of this association may depend on the characteristics of the decision-making situation, highlighting the importance of considering behavioural context when examining personality–risk relationships.

The most unexpected finding concerned Conscientiousness. Contrary to the expected general direction, higher Conscientiousness was not consistently associated with a lower probability of selecting the risky option across the four scenarios. Instead, in the BETA and DELTA loss lotteries, higher Conscientiousness was associated with greater odds of choosing the risky option. Although this finding was not predicted a priori, previous research suggests that associations between Conscientiousness and risk-taking are not uniform across behavioural domains (Weller & Tikir, 2011), supporting the view that personality–risk relationships may depend on the characteristics of the decision context. A conceptually related finding was reported by Balasuriya et al. (2025), who observed that individuals higher in Conscientiousness took greater investment risks following poorer prior financial performance. The authors suggested that highly conscientious individuals may be motivated to take riskier actions in an effort to recover from setbacks and achieve their goals. Similarly, in the present study, the risky option in the loss lotteries offered an opportunity to avoid a certain negative outcome and may therefore have been perceived as a goal-directed strategy to prevent a guaranteed loss rather than as an expression of impulsivity or poor self-control. Nevertheless, this interpretation remains tentative. Balasuriya et al. examined responses to experienced outcomes in a sequential investment simulation, whereas the present study involved independent hypothetical lottery choices. Moreover, the current study did not assess participants’ reasoning, perceived control, deliberation, or subjective interpretation of the alternatives. Therefore, the association between Conscientiousness and risky choice in loss lotteries should be regarded as preliminary and examined in independent samples before broader theoretical conclusions are drawn.

The findings have several implications, although they should be formulated cautiously. First, the response patterns observed in the four hypothetical lotteries are consistent with prospect-theory predictions concerning choices involving gains and losses. Second, the results suggest that incorporating the HEXACO model into research on hypothetical lottery choices may be useful for examining whether selected traits show different associations across decision scenarios. Third, the findings support the value of analyzing individual differences in relation to decision context rather than treating personality traits exclusively as isolated, context-independent predictors. These findings may also have practical implications for financial education by highlighting the importance of teaching individuals how gain–loss framing can systematically influence financial decisions. Similarly, financial counselling may benefit from recognizing that risk preferences are not necessarily stable across situations but may depend on the interaction between personality characteristics and the specific decision context. From a behavioural finance perspective, the present results support approaches that integrate both contextual and dispositional factors when explaining financial decision-making. However, the present evidence is not sufficient to establish HEXACO traits as determinants of financial risk taking or stable risk preferences. It identifies preliminary, context-dependent associations within the four lottery scenarios that require replication using broader and psychometrically stronger measures.

Several limitations should be considered. First, the study assessed stated choices in hypothetical financial lotteries, and the findings cannot be assumed to generalize to decisions involving real financial consequences. Although hypothetical lotteries are commonly used in decision-making research, the absence of incentive-compatible outcomes limits the extent to which the results can be generalized to real financial behaviour. Second, only four decision scenarios were examined. Although this design captured basic differences between gains and losses and between small and large stakes, each condition was represented by a single lottery. Therefore, it is not possible to determine whether the observed effects reflect stable responses to gain and loss contexts more generally or responses to the specific wording, amounts, and probabilities used in these particular scenarios. Accordingly, the four choices should not be regarded as a comprehensive or psychometrically robust measure of financial risk taking. They represent participants’ responses to four specific hypothetical scenarios, each defined by a particular combination of outcome domain, monetary amount, probability, and wording. Moreover, the safe and risky options were not matched with respect to expected value. Consequently, participants’ choices may have reflected responses not only to outcome uncertainty but also to differences in expected value. Future studies should consider using lotteries with equivalent expected values to better isolate preferences for risk from responses to expected outcomes. Third, two of the lotteries involved very large monetary amounts, namely PLN 300,000 and PLN 400,000. Such amounts may have been psychologically salient, but they are unlikely to reflect typical financial experiences or decisions faced by most participants and may have been difficult for some participants to evaluate realistically. This may limit the ecological validity of the findings. Fourth, the study was cross-sectional and observational with respect to the associations between personality traits and risky choice. The results should therefore be interpreted as associations rather than evidence of causal relationships. Although the sample was diverse with respect to age, gender, education, and financial situation, the present analyses focused on personality traits and lottery type and did not systematically examine the role of these demographic covariates. Consequently, their omission may have resulted in residual confounding, and future research should examine whether the observed associations remain robust after controlling for these characteristics. Fifthly, another limitation is that the present study relied exclusively on four hypothetical lottery scenarios and did not include additional validated behavioural measures of financial risk taking (e.g., DOSPERT, Holt–Laury task, BART, or the Columbia Card Task). Consequently, it cannot be determined whether the observed associations generalize to broader measures of financial or behavioural risk taking. Future studies should combine multiple experimental paradigms and validated behavioural measures to evaluate the robustness and generalizability of the observed context-dependent associations. Finally, the observed interaction effects, especially the positive association between Conscientiousness and risky choice in loss lotteries, were not independently replicated and should be treated as preliminary. Future research should use a broader range of decision tasks, include multiple scenarios within each condition, consider choices involving real or incentive-compatible financial consequences, and examine whether the context-dependent associations observed in the present study replicate in other samples.

In summary, risky choice in financial lotteries depended strongly on decision context. Participants were more likely to choose the safe option in gain conditions and the risky option in loss conditions, in line with prospect theory. Honesty–Humility showed a significant overall association with the probability of selecting the risky option across the four scenarios, whereas the remaining HEXACO dimensions did not. In addition, Honesty–Humility and Conscientiousness showed significant interactions with lottery condition. Higher Honesty–Humility was associated with lower odds of risky choice in gain lotteries, whereas higher Conscientiousness was associated with higher odds of risky choice in loss lotteries. These findings provide preliminary evidence that associations between selected personality traits and the probability of choosing the risky option may differ across specific hypothetical lottery scenarios. Because the evidence is based on only four hypothetical lottery scenarios and lacks independent replication, the results should be interpreted cautiously. They should not be treated as evidence of stable financial risk preferences or generalized to financial risk taking more broadly.

## Figures and Tables

**Table 1:** Distribution of safe and risky choices across lottery conditions: ALFA, BETA, GAMMA, and DELTA.

Lottery and Choice (0—Safe Option, 1—Risky Option)	N	%
ALFA		
0	231	74.5
1	79	25.5
BETA		
0	141	45.5
1	169	54.5
GAMMA		
0	247	79.7
1	63	20.3
DELTA		
0	129	41.6
1	181	58.4

Note: 0 = safe choice; 1 = risky choice. ALFA = small gain; BETA = small loss; GAMMA = large gain; DELTA = large loss.

**Table 2:** GLMM fit statistics for the effect of lottery type.

Deviance	log Lik.	df	AIC	BIC
930.253	−700.102	5	1410.204	1435.819

**Table 3:** Effect of lottery type on risky choice in the GLMM (ALFA as the reference category).

	Fixed Effects Significance Test (χ^2^, Type III)			Fixed Effects Estimation in the GLMM Model (ALFA= Reference)				
Predictor	df	χ^2^	*p* (Omnibus)	b (Logit)	SE	t	*p*(b)	OR = exp(b)
Lottery	3	206.795	<0.001	-	-	-	-	-
Intercept (ALFA)	-	-	-	−1.588	0.196	−8.109	<0.001	0.20
BETA vs. ALFA	-	-	-	1.862	0.226	8.249	<0.001	6.44
GAMMA vs. ALFA	-	-	-	−0.411	0.228	−1.802	0.072	0.66
DELTA vs. ALFA	-	-	-	2.100	0.231	9.101	<0.001	8.17

Note: ALFA was the reference category. Coefficients for BETA, DELTA, and GAMMA refer to differences from ALFA. OR was calculated as exp(b). OR > 1 indicates higher odds of choosing the risky option relative to ALFA, while OR < 1 indicates lower odds of choosing the risky option relative to ALFA.

**Table 4:** GLMM fit statistics for the model including HEXACO traits and lottery type.

Deviance	log Lik.	df	AIC	BIC
863.696	−672.297	29	1402.593	1551.157

**Table 5:** Main effects of HEXACO traits and lottery type in the GLMM predicting risky choice.

	Fixed Effects Significance Test (χ^2^, Type III)				Fixed Effects Estimation in the GLMM (ALFA = Reference)				
Predictor	df	χ^2^	*p* (Omnibus)	*p* FDR	b (Logit)	SE	t	*p*(b)	OR = exp(b)
Intercept	-	-	-		−1.717	0.211	−8.140	<0.001	0.18
Lottery	3	215.018	<0.001		-	-	-	-	-
Honesty-Humility	1	8.131	0.004	0.024	−0.632	0.230	−2.754	0.006	0.53
Emotionality	1	2.182	0.140	0.420	−0.301	0.211	−1.425	0.154	0.74
Extraversion	1	0.343	0.558	0.739	0.050	0.217	0.232	0.817	1.05
Agreeableness	1	0.111	0.739	0.742	0.017	0.202	0.083	0.934	1.02
Conscientiousness	1	0.254	0.614	0.739	−0.148	0.227	−0.653	0.514	0.86
Openness to Experience	1	0.882	0.348	0.696	−0.156	0.207	−0.754	0.451	0.86

Note: ALFA was used as the reference category for the Lottery factor. The b coefficients refer to the main effects of predictors in the GLMM while controlling for lottery type. Continuous predictors were standardized; therefore, b and OR values refer to a one standard deviation increase in the predictor. OR was calculated as exp(b). OR > 1 indicates higher odds of choosing the risky option, whereas OR < 1 indicates lower odds of choosing the risky option. The *p* FDR column reports *p*-values after Benjamini–Hochberg correction.

**Table 6:** Interactions between HEXACO dimensions and lottery type in the GLMM.

Effect	χ^2^	df	*p*	*p* FDR
Honesty-Humility × Lottery	10.891	3	0.012	0.036
Emotionality × Lottery	4.380	3	0.223	0.448
Extraversion × Lottery	0.193	3	0.979	0.979
Agreeableness × Lottery	0.633	3	0.889	0.979
Conscientiousness × Lottery	11.328	3	0.010	0.036
Openness to Experience × Lottery	3.169	3	0.366	0.551

Note: The table presents tests of interactions between individual HEXACO dimensions and lottery type. Each interaction tests whether the association between a given personality trait and risky choice differs depending on lottery type. Continuous predictors were standardized. The *p* FDR column reports *p*-values after Benjamini–Hochberg correction.

**Table 7:** Simple effects of Honesty–Humility and Conscientiousness on risky choice across lottery conditions.

Predictor	Lottery	b	SE	t	*p*	*p* FDR	OR	OR Interpretation
Honesty–Humility	ALFA	−0.670	0.200	−3.347	<0.001	0.004	0.51	approximately 49% lower odds for a 1 SD increase in the predictor
Honesty–Humility	BETA	0.345	0.176	1.958	0.050	0.067	1.41	not significant after FDR; directionally approximately 41% higher odds for a 1 SD increase in the predictor
Honesty–Humility	GAMMA	−0.474	0.205	−2.317	0.021	0.034	0.62	approximately 38% lower odds for a 1 SD increase in the predictor
Honesty–Humility	DELTA	0.228	0.176	1.298	0.194	0.222	1.26	not significant; directionally approximately 26% higher odds for a 1 SD increase in the predictor
Conscientiousness	ALFA	−0.397	0.195	−2.034	0.042	0.067	0.67	not significant after FDR; directionally approximately 33% lower odds for a 1 SD increase in the predictor
Conscientiousness	BETA	0.602	0.181	3.331	<0.001	0.004	1.83	approximately 83% higher odds for a 1 SD increase in the predictor
Conscientiousness	GAMMA	−0.232	0.204	−1.139	0.255	0.255	0.79	not significant; directionally approximately 21% lower odds for a 1 SD increase in the predictor
Conscientiousness	DELTA	0.523	0.180	2.905	0.004	0.011	1.69	approximately 69% higher odds for a 1 SD increase in the predictor

Note: Simple effects were estimated by re-estimating the same GLMM with successive levels of the Lottery factor set as the reference category. Coefficients refer to the effect of a given predictor within a specific lottery condition. Continuous predictors were standardized; therefore, b and OR values refer to a one standard deviation increase in the predictor. OR = odds ratio, calculated as exp(b). OR < 1 indicates lower odds of risky choice, whereas OR > 1 indicates higher odds of risky choice. The *p* FDR column reports *p*-values after false discovery rate correction for multiple testing of simple effects.

**Table 8:** Estimated predicted probabilities of choosing the risky option across lottery conditions for the significant HEXACO interactions.

Predictor	Lottery	−1 SD	Mean	+1 SD
Honesty–Humility	ALFA	0.253 (0.161–0.372)	0.152 (0.106–0.214)	0.087 (0.047–0.155)
	BETA	0.557 (0.426–0.682)	0.572 (0.485–0.654)	0.586 (0.454–0.706)
	GAMMA	0.166 (0.099–0.264)	0.108 (0.072–0.158)	0.068 (0.036–0.126)
	DELTA	0.622 (0.490–0.737)	0.628 (0.543–0.706)	0.634 (0.504–0.747)
Conscientiousness	ALFA	0.172 (0.103–0.275)	0.152 (0.106–0.214)	0.134 (0.077–0.223)
	BETA	0.438 (0.314–0.571)	0.572 (0.485–0.654)	0.695 (0.572–0.796)
	GAMMA	0.114 (0.064–0.196)	0.108 (0.072–0.158)	0.101 (0.056–0.176)
	DELTA	0.489 (0.360–0.620)	0.628 (0.543–0.706)	0.748 (0.632–0.837)

Note. Values represent estimated predicted probabilities obtained from the generalized linear mixed-effects model on the response scale. Values in parentheses are 95% confidence intervals. Low and high levels of each personality trait correspond to one standard deviation below and above the sample mean, respectively.

## Data Availability

The anonymized dataset generated and analyzed during the current study, together with a codebook describing the variables, is available in the Open Science Framework repository: https://osf.io/hr7x2/files/osfstorage (accessed on 7 August 2026).
